# Inhibitory effects of metformin at low concentration on epithelial–mesenchymal transition of CD44^+^CD117^+^ ovarian cancer stem cells

**DOI:** 10.1186/s13287-015-0249-0

**Published:** 2015-12-30

**Authors:** Rongrong Zhang, Ping Zhang, Hong Wang, Dongming Hou, Wentao Li, Guishan Xiao, Chenwei Li

**Affiliations:** Department of Gynecology, Xinhua Hospital, School of Medicine, Shanghai Jiao Tong University, Shanghai, 200092 China; Department of Otolaryngology & Head and Neck Surgery, Xinhua Hospital, School of Medicine, Shanghai Jiao Tong University, Shanghai, 200092 China; Department of Interventional Radiology, Cancer Hospital, Fudan University, Shanghai, 200032 China; School of Pharmaceutical Science and Technology, Dalian University of Technology, Dalian, 116024 China; Sunstem Biotechnology Co., Ltd, Shanghai, 200439 China

**Keywords:** Metformin, Epithelial–mesenchymal transition, Ovarian cancer stem cells

## Abstract

**Background:**

Although metformin, a first-line drug for treating diabetes, may play an important role in inhibition of epithelial ovarian cancer cell growth and cancer stem cells (CSCs), metformin at low dose showed less effect on the proliferation of ovarian cancer cells. In this study, we evaluated the effect of metformin at low dose on ovarian CSCs in order to understand the molecular mechanisms underlying.

**Methods:**

The inhibitory effects of metformin at los dose on proliferation and population of ovarian cancer cells including SKOV3 and A2780 were assessed by cell proliferation assay and flow cytometry. Quantitative real-time PCR assay on expression of Bcl-2, Survivin and Bax was performed to determine the effect of metformin at low dose on epithelial-mesenchymal transition (EMT) of cancer cells and CSCs. Tumor sphere formation assay was also performed to evaluate the effect of metformin on spheres forming ability of CSCs. The therapeutic efficacy and the anti-CSC effects of metformin at low dose were investigated by using both SKOV3 cells and primary tumor xenografts. In addition, the CSC frequency and EMT in tumor xenograft models were also assessed by flow cytometry and quantitative real-time PCR.

**Results:**

Metformin at low dose did not affect the proliferation of ovarian cancer cells. However, it inhibited population of CD44^+^CD117^+^ selectively, neither CD133^+^ nor ALDH^+^ cells. It suppressed expression of snail2, twist and vimentin significantly in cancer cells and CD44^+^CD117^+^ CSCs *in vitro*. Low dose of metformin reduced survivin expression in CSCs. Low concentrations of metformin inhibited the secondary and the tertiary tumor sphere formation, decreased SKOV3 and primary ovarian tumor xenograft growth, enhanced the anticancer effect of cisplatin, and lowered the proportion of CD44^+^CD117^+^ CSCs in the xenograft tissue. Metformin was also associated with a reduction of snail2, twist, and vimentin in CD44^+^CD117^+^ ovarian CSCs *in vivo*.

**Conclusions:**

Our results implicate that metformin at low dose inhibits selectively CD44^+^CD117^+^ ovarian CSCs through inhibition of EMT and potentiates the effect of cisplatin.

## Background

Ovarian cancer is the fifth most common type of cancer in females and the leading cause of mortality for gynecological malignancies. Epithelial ovarian cancer (EOC) accounts for more than 90 % of all ovarian neoplasms [1, 2].

To understand the exact biological features of ovarian carcinoma, series of new studies are being focused on cancer stem cells (CSCs). Study of CSCs suggests that these are a rare population of cancer cells with inherent chemoresistance, capable of regeneration of the various cell types within tumors, which then leads to relapse of therapy [3, 4]. Drugs targeting CSCs may offer great promise for the development of novel anticancer drugs. Highly diversified phenotypes in ovarian CSCs have been found. Well-studied cell surface biomarkers including CD44/CD117, CD133, and aldehyde dehydrogenase (ALDH) are used extensively to identify ovarian CSCs [5–11].

Metformin, a traditional medication for type 2 diabetes, has been demonstrated to have antiproliferative and proapoptotic effects on ovarian cancer [12], and has been used to target CSCs in ovarian cancer [6]. Recent studies indicated that metformin can inhibit the growth and the proliferation of ovarian CSCs in vitro and in vivo. These results provide a rationale for using metformin to treat patients with ovarian cancer [6].

The epithelial–mesenchymal transition (EMT) is a process by which an epithelial cancer cell loses its cell polarity and cell–cell adhesion and acquires the capacity to migrate and metastasize. Epithelial cancer cells through EMT can undergo a phenotypic switch, which allows these polarized and immobile epithelial cells to become motile mesenchymal cells [13–15]. EMT has been linked to the ability for self-renewal and generation of multiple lineages [14]. Although the latest studies showed that EMT can initiate regeneration of CSCs from immortalized human mammary epithelia [14, 16, 17], the molecular mechanism underlying regeneration of CSCs through EMT is unknown.

A series of studies has shown that metformin may repress the EMT transcriptionally and repress a cell phenotype associated with CSCs in breast cancer [18, 19]. It also inhibited cellular transformation and killed breast CSCs selectively in vitro and in vivo [19]. Snail1 and snail2 are key transcriptional factors to regulate progression, functionality, and survival of metastatic ovarian stem cancer cells. Activation of Snail1 and Snail2 results in regeneration of stem-like cells through EMT, causing cellular resistance to the therapy and failure of p53-mediated apoptosis, and leading to activation of a self-renewal program [20].

Although the dose of metformin used in the clinic is much lower than that used in most research, metformin at a dose less than 1 mM shows less effect on proliferation of ovarian cancer cells in vitro [6]. However, a recent study of pancreatic cancer showed that metformin at low concentrations selectively inhibited the proliferation and the invasive capacities of CD133^+^ cells in vitro, and of pancreatic cancer xenograft growth in vivo [21]. Recently, data from a study suggested that metformin at low dose might transform ovarian cancer cells or CSCs into noncancerous cells by reprogramming CSCs, leading to benefit in patients who had these cancers with minimal side effects [22].

In this study, we hypothesized that metformin at low dose inhibits growth of ovarian cancer cells, and found that metformin at low dose inhibited proliferation of ovarian CSCs both in vitro and in vivo, suggesting a novel mechanism underlying inhibitory effects of metformin on ovarian CSCs.

## Methods

### Cell culture

The human ovarian cancer cell lines SKOV3 and A2780 were obtained from the Cell Bank of the Shanghai Institute of Biochemistry & Cell Biology, Shanghai Institute for Biological Sciences, Chinese Academy of Sciences, Shanghai, China (http://www.cellbank.org.cn), and were cultured in RPMI-1640 medium supplemented with 10 % fetal bovine serum (Gibco, Grand Island, NY, USA). All cell lines were maintained in a humidified atmosphere at 37 °C with 5 % CO_2_. For tumor spheres culture in vitro, cells were plated in ultralow attachment six-well plates (Corning Inc., Corning, NY, USA) at a density of 10,000 cells per well in serum-free endothelial basal medium-2 (Lonza, Basel, Switzerland) with 10 ng/ml leukemia inhibitory factor (Sigma. St. Louis, MO, USA), 20 ng/ml human fibroblast growth factor-2 (Sigma. St. Louis, MO, USA), or 20 ng/ml epidermal growth factor (Gibco, Grand Island, NY, USA). Sphere cultures were passaged every 7–10 days. To passage spheres, media were removed and spheres were incubated at room temperature for 5 minutes in 0.05 % trypsin (Gibco, Grand Island, NY, USA). Spheres were observed under the microscope to verify dissociation. Cells were then washed with Hanks’ buffered salt solution (HBSS; Gibco) and filtered through a 40 nm strainer before replanting.

### Cell proliferation assays

Cells were cultured at a density of 3 × 10^3^ cells per well in 96-well plates in RPMI-1640 media with 10 % fetal bovine serum (FBS). After 24 hours, Cell Titer 96H Aqueous One Solution Reagent (Promega, Madison, WI, USA) was added to each well according to the manufacturer’s instructions. After culture for 2 hours, cell viability was determined by measuring the absorbance at 490 nm using a Bio-Rad plate-reader (Hercules, California, CA, USA).

### Quantitative real-time PCR

cDNA was first synthesized using equivalent amounts of total RNA (0.5-1 μg) with random primers in a 20 μl reverse-transcriptase reaction mixture (Promega). Real-time quantitative RT-PCR (Taqman) primers for snail1, snail2, twist, vimentin, E-cadherin, and β-actin were designed and purchased from Applied Biosystems as Assay-on-Demand™ Gene Expression Products. Real-time RT-PCR was performed following the supplier’s directions. The 20 μl PCR mixture contained 10 μl of 2× Taqman universal PCR master mixes, 1 μl of 20× working stock of expression assay mix, and 50 ng RNA converted DNA. Real-time PCR was performed in an ABI PRISM 7900HT sequencing detection system (Applied Biosystems, Foster City, CA, USA). The assay for each sample was performed in triplicate. Fluorescence of the PCR products was detected using same apparatus. The number of cycles required for the amplification plot to reach the threshold limit, the Ct value, was used for quantification.

### Western blotting assay

Immunoblot analysis was performed using antibodies against snail1, snail2, twist, vimentin, E-cadherin, and β-actin (Cell Signaling Technology, Beverly, MA, USA) diluted according to the manufacturers’ instructions. After analysis, the blots were stripped, washed, and reprobed with β-actin antibody to serve as a loading control. Protein expression was quantified using a Kodak Gel Documentation System (model 1D 3.6; Kodak, Rochester, NY, USA).

### Preparation for single cell suspensions of tumor cells

Before digestion with collagenase, xenograft tumors were cut up into small pieces with scissors, and then minced completely using sterile scalpel blades. To obtain single cell suspensions, the resultant minced tumor pieces were mixed with ultra-pure collagenase IV (Worthington Biochemical, Lakewood, NJ, USA) in medium 199 (200 units of collagenase per ml) and allowed to incubate at 37 °C for 2.5–3 hours for enzymatic dissociation. The specimens were further mechanically dissociated every 15–20 minutes by pipetting with a 5 ml pipette. At the end of the incubation, cells were filtered through a 40 μm nylon mesh and washed with HBSS/20 % FBS, and then washed twice with HBSS.

### Flow cytometry

Dissociated cells were counted and transferred to a 5 ml tube, washed twice, and resuspended in HBSS with 2 % FBS at concentrations of 10^6^ cells per 100–200 μl. Sandoglobin solution (1 mg/ml) was then added to the sample at a dilution of 1:20 and the sample was then incubated on ice for 20 minutes. The sample was then washed twice and resuspended with HBSS containing 2 % FBS. Antibodies were incubated for 20 minutes on ice, and the sample was washed twice with HBSS containing 2 % FBS. When needed, a secondary antibody was followed by a 20-minute incubation. After another washing, cells were resuspended in HBSS buffer containing 4′,6-diamidino-2-phenylindole (DAPI; 1 μg/ml). The antibodies used were anti-CD44 allophycocyanin (APC), anti-CD117 (PE), anti-CD133 (FITC), and anti-H2K (PharMingen, Franklin Lakes, NJ, USA), each at a dilution of 1:40. In all experiments using human xenograft tissue, infiltrating mouse cells were eliminated by discarding H2K (mouse histocompatibility class I) cells during flow cytometry. Dead cells were eliminated using the viability dye DAPI. ALDH enzymatic activity was assessed using the ALDEFLUOR kit as per the manufacturer’s protocol (Stem Cell Technologies, Vancouver, BC, Canada). Cells treated with ALDH inhibitor diethylaminobenzaldehyde (DEAB; 50 mM/l) were used as control. Flow cytometry was performed on a FACS Aria machine (BD Immunocytometry Systems, Franklin Lakes, NJ, USA). Side scatter and forward scatter profiles were used to eliminate cell doublets. Cells were routinely sorted twice, and the cells were reanalyzed for purity, which typically was greater than 97 %.

### Analysis of cell cycle analysis

For cell cycle analysis by flow cytometry, cells were fixed with 70 % ethanol overnight at 4 °C. Cell pellets were then suspended in 300 μl phosphate-buffered saline (PBS) containing 100 μg/ml RNase for 30 minutes at room temperature, and then 10 μg/ml propidium iodide (Calbiochem, San Diego, CA, USA) were added. The distribution of cells in the different phases of the cell cycle was analyzed from DNA histograms using BD Cell Quest software (Becton Dickinson Biosciences, San Diego, CA, USA).

### Subcutaneous implantation of cells in NOD-SCID mice

Single cell suspensions (5 × 10^5^ cells) were made with serum-free RPMI/Matrigel (BD Bioscience, San Jose, CA) mixture (1:1 volume) and injected subcutaneously into the right and left mid abdominal area of 8-week-old male nude mice using a 27-gauge needle. Mice obtained from Shanghai SLAC Lab. Animal Co., Ltd (Shanghai, China) were monitored daily for tumor growth. The treatments were started when the tumor size reached approximately 150 mm^3^, and then mice received no treatment, metformin 20 mg/kg intraperitoneally (i.p.) daily, cisplatin 250 μg/kg i.p. for 3 days, or cisplatin 250 μg/kg i.p. plus metformin 20 mg/kg i.p. daily. Tumor growth was measured using calipers, and animals underwent autopsy at 4 and 9 weeks after treatment and tumor growth was assessed. All experiments in this study were approved by the local institutional review board and ethics committee at the authors’ affiliated institutions and animal experiments were carried out in accordance with the established institutional guidelines for animal care and use of Laboratory Animals of the Shanghai Jiao Tong University (Shanghai, China).

### Statistical analysis

Data are expressed as the mean ± standard error. Statistically significant differences were determined by the unpaired Student’s *t* test between two groups and one-way analysis of variance followed by Dunnett’s test between multiple comparisons. *P* <0.05 was considered significant.

## Results

### Metformin at low dose decreased CD44^+^CD117^+^ CSCs

**Fig. 1:**
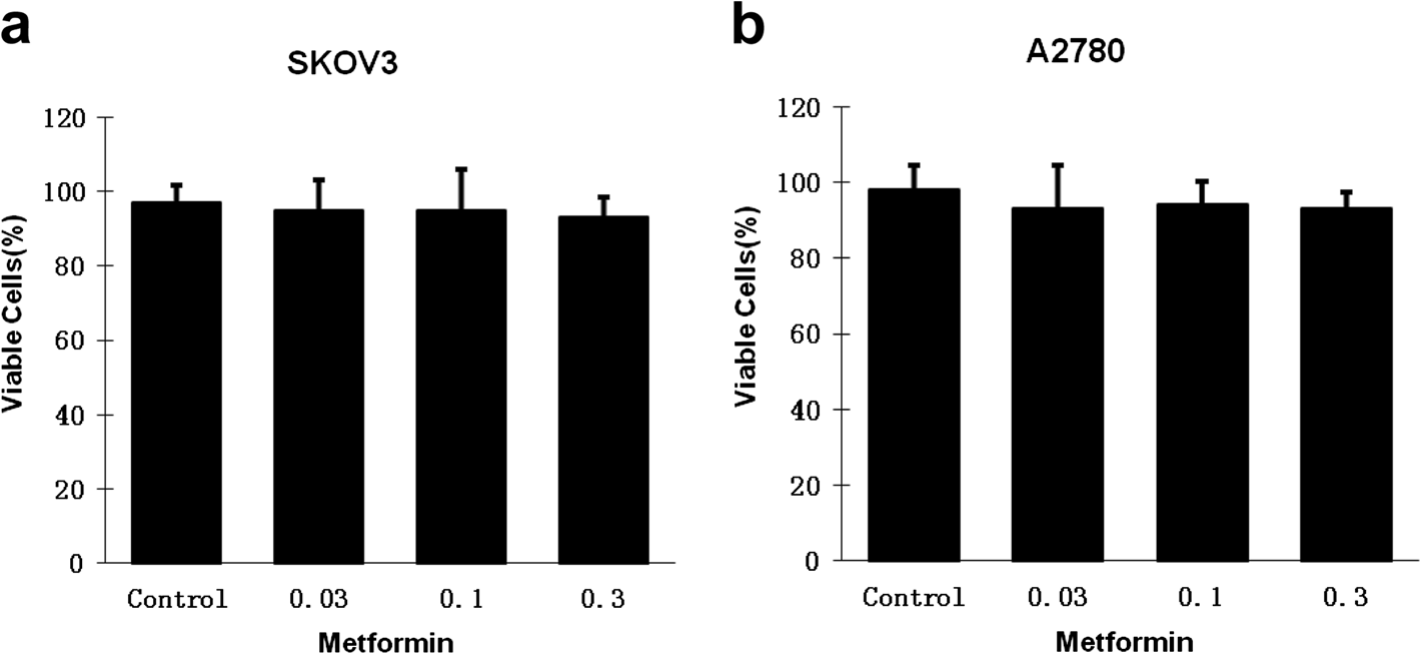
Low concentrations of metformin did not inhibit the proliferation of pancreatic cancer cells. SKOV3 (**a**) and A2780 (**b**) cells were incubated with different concentrations of metformin for 72 hours and numbers of viable cells were determined by cell proliferation assay

**Fig. 2:**
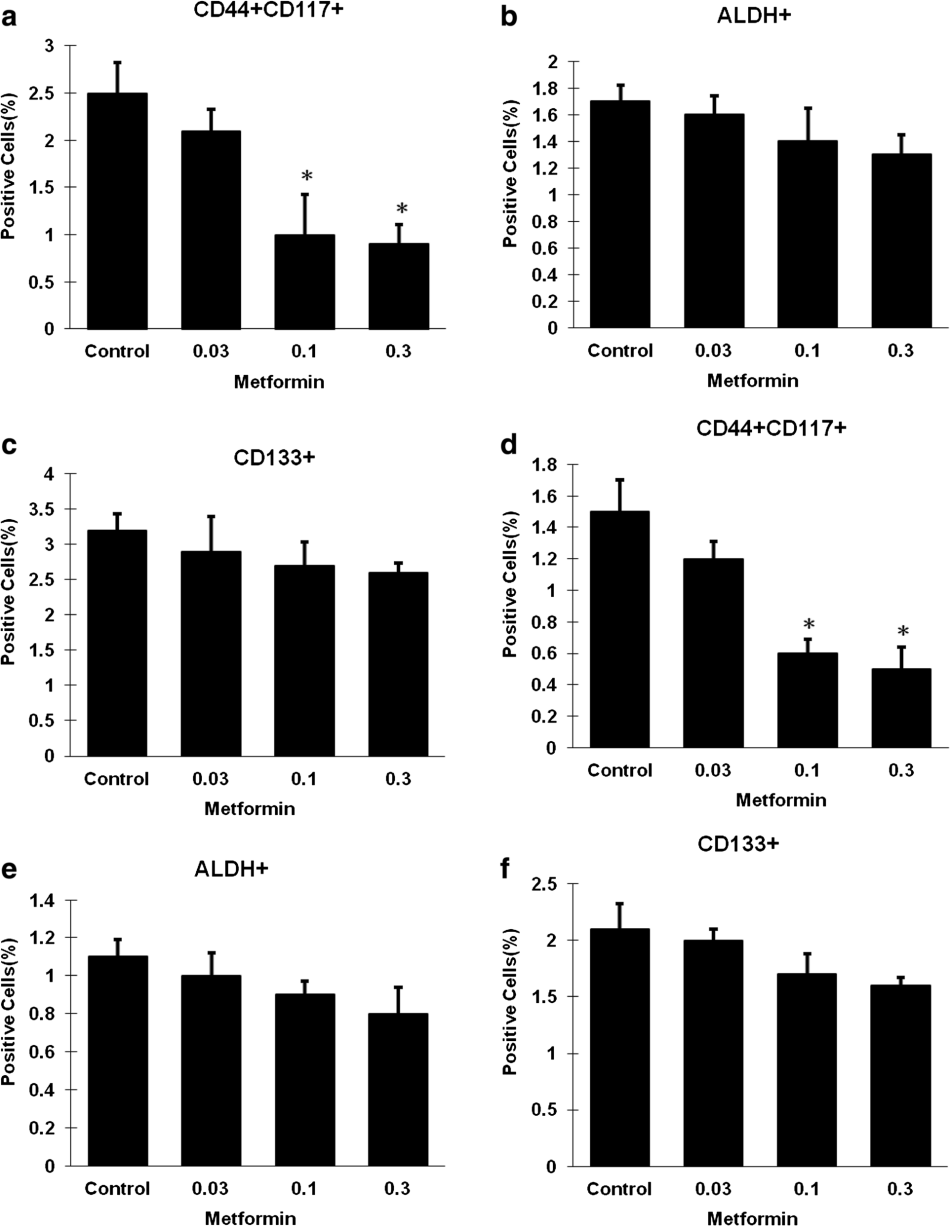
Low concentrations of metformin selectively decreased the CD44^+^CD117^+^ ovarian cancer cell population. SKOV3 (**a**, **b**, **c**) and A2780 (**d**, **e**, **f**) cells were incubated with different concentrations of metformin for 72 hours, and the proportions of cells expressing different CSC surface markers were determined by flow cytometry. For both cell lines, the proportions of CD133^+^ and ALDH^+^ were not altered by treatment with low concentrations of metformin (0.03–0.3 mM) (**b**, **c**, **e**, **f**), but the proportion of CD44^+^CD117^+^cells was reduced in a dose-dependent manner (**a**, **d**). *ALDH* aldehyde dehydrogenase. * Significant difference compared with control (*P* < 0.05)

Metformin at a dose range of 0.03-0.3 mM showed no significant inhibitory effect on two ovarian cancer cell lines (Fig. 1). Flow cytometry assay was performed by analysis of the percentage of ALDH^+^, CD133^+^, and CD44^+^CD117^+^ cells in SKOV3 and A2780 cells treated with metformin at a dose range of 0.03–0.3 mM for 72 hours. Metformin at low dose resulted in a 2.5-fold decrease in the CD44^+^CD117^+^ CSC population at a metformin dose of 0.1 mM and in a 2.8-fold decrease at a dose of 0.3 mM in SKOV3 cells (Fig. 2a). Metformin caused a 2.5-fold decrease in the CD44^+^CD117^+^ CSC population at a dose of 0.1 mM and a threefold decrease at 0.3 mM in A2780 cells (Fig. 2d). However, metformin showed no inhibitory effect on both CD133^+^ and ALDH^+^ subpopulations in the dose range of 0.03–0.3 mM (Fig. 2b, c, e, f).

### Metformin inhibited both EMT of SKOV3 cells and EMT of CD44^+^CD117^+^ CSCs

**Fig. 3:**
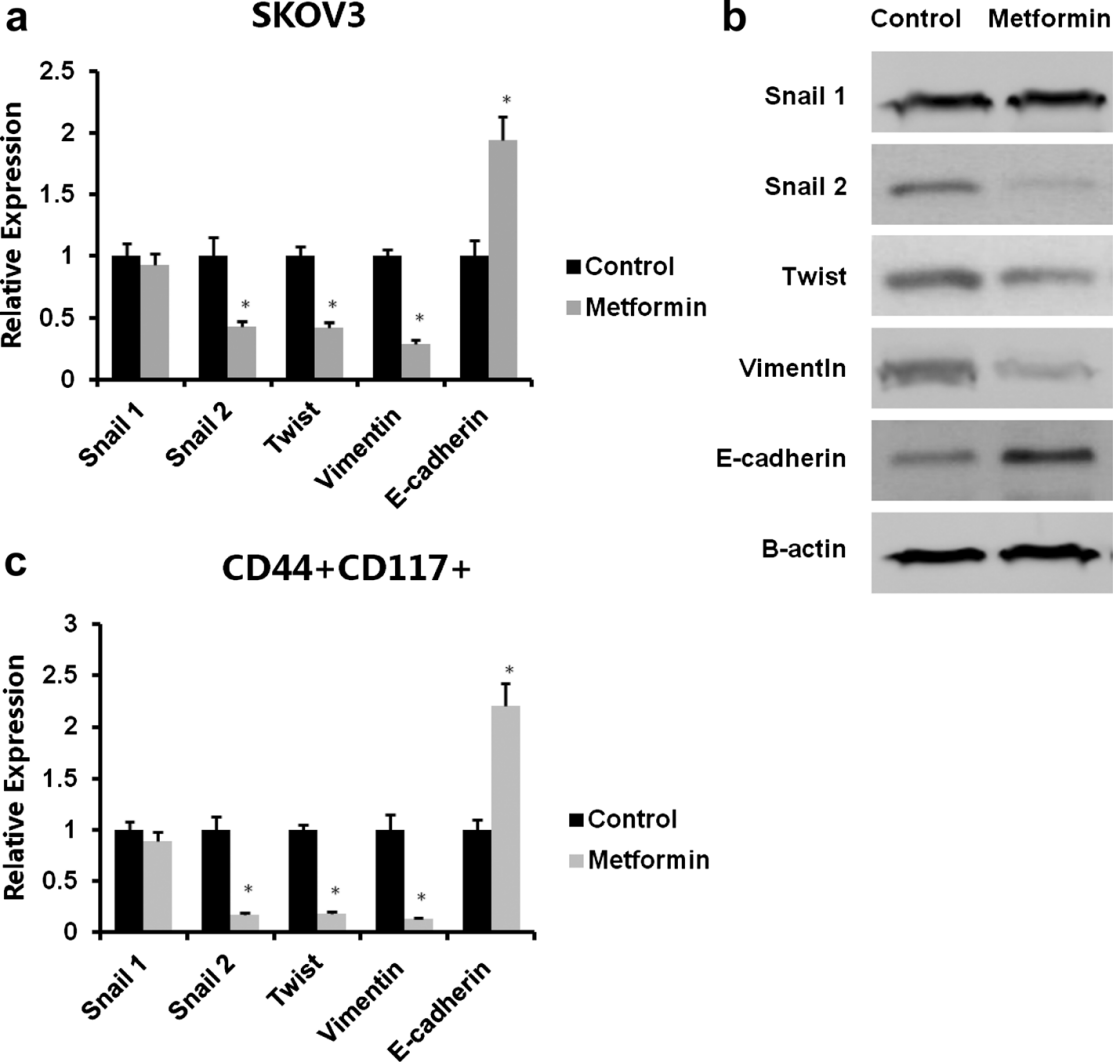
Low concentrations of metformin reduced EMT of ovarian cancer cells and the CD44^+^CD117^+^ CSC population. SKOV3 cells were incubated with 0.1 mM metformin for 72 hours. The CD44^+^CD117^+^ cells were isolated by flow cytometry. Total RNA was isolated and mRNA was quantitated by real-time RT-PCR. mRNA expression of snail1, snail2, twist, vimentin, and E-cadherin was analyzed. Data show that treatment with 0.1 mM metformin reduced snail2, twist, and vimentin, but not snail1expression level, and upregulated the E-cadherin expression level (**a**). Bands detected with antibodies indicate the immune reactivity for snail1, snail2, twist, vimentin, E-cadherin, and β-actin: 72 hours of treatment with 0.1 nM metformin significantly downregulated snail2, twist, and vimentin, but not snail1, and upregulated E-cadherin as shown by western blotting (**b**). Total RNA was isolated from CD44^+^CD117^+^ cells and the mRNA was quantitated by real-time RT-PCR. Metformin at 0.1 mM significantly reduced snail2, twist, and vimentin, but not snail1 expression level, and upregulated the E-cadherin expression level (**c**). * Significant difference compared with control (*P* < 0.05)

To investigate the effect of metformin at low dose (0.1 mM) on the EMT of SKOV3 cells, SKOV3 cells were treated with metformin at 0.1 mM for 72 hours and expression of EMT components, including snail1, snail2, twist, vimentin, and E-cadherin, was then analyzed by quantitative real-time PCR. We observed that metformin at 0.1 mM showed no effect on snail1 expression, a 2.3-fold decrease on snail2, a 2.4-fold decrease on twist, a 3.4-fold decrease on vimentin, and a 1.9-fold increase on E-cadherin (Fig. 3a). Western blotting assay showed that metformin increased significantly the expression of E-cadherin and decreased snail2, twist, and vimentin, which were consistent with the quantitative RT-PCR results (Fig. 3b). To determine expression of the EMT biomarkers, flow cytometry was used to isolate CD44^+^CD117^+^ cells. Data from quantitative real-time PCR showed that metformin at 0.1 mM caused no change in snail1 expression, but a 5.9-fold decrease for snail2, a 5.6-fold decrease for twist, a 7.7-fold decrease for vimentin, and a 2.32-fold increase for E-cadherin (Fig. 3c).

### Metformin reduced the CD44^+^CD117^+^ subpopulation of ovarian CSCs by inhibition of its self-renewal

**Fig. 4:**
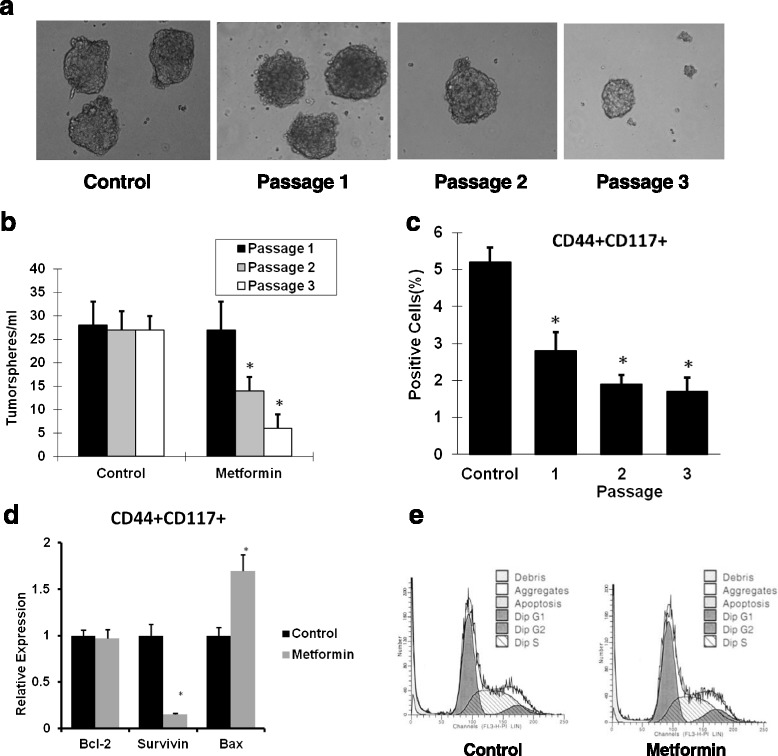
Metformin inhibits CSCs in tumor spheres and regulates their self-renewal. Isolated CD44^+^CD117^+^ cancer cells were cultured in sphere medium for 24 hours and then incubated with or without 0.1 mM metformin. Seven days of treatment with 0.1 mM metformin failed to inhibit tumor sphere formation. Tumor spheres were then dissociated with 0.05 % trypsin and cultured in ultralow plates with 0.1 mM metformin. Metformin at 0.1 mM inhibits CD44^+^CD117^+^ cells forming spheres with serial passaging (**a**, **b**). Metformin at 0.1 mM also reduced the CD44^+^CD117^+^ cell population with serial passaging (*n* = 3) (**c**). Metformin at 0.1 mM significantly reduced Survivin but not Bcl-2 expression level, and upregulated Bax expression level in CD44^+^CD117^+^ cells (**d**). Representative cell cycle histograms following flow cytometric analysis of propidium iodide (*PI*)-stained CD44^+^CD117^+^ cells show no change in the percentage of cells entering the S phase upon 0.1 nM metformin treatment (**e**). * Significant difference compared with control (*P* < 0.05)

To study whether metformin inhibits subpopulation of ovarian cancer cells, CD44^+^CD117^+^ SKOV3 cells were sorted and grown in the presence or absence of metformin. The results showed that metformin at 0.1 mM decreased the CD44^+^CD117^+^ cell population inside tumorspheres significantly, as shown in Fig. 4c. To determine the inhibitory effects of metformin on self-renewal of CSCs, sorted CSCs were treated with metformin at 0.1 mM. The results showed that first-passage sphere formation was not significantly different regardless of metformin at 0.1 mM. However, the significant inhibitory effects of metformin at 0.1 mM on formation of new tumorspheres was observed as passaging went on. Cells at passage 3 treated with metformin at 0.1 mM showed an approximately fourfold decrease in their ability to form new tumor spheres as compared with controls (Fig. 4a, b).

### Metformin at low dose inhibited proliferation of CD44^+^CD117^+^ cells by significantly reducing Survivin expression

To study whether metformin affects proliferation of the subpopulation of CD44^+^CD117^+^ cells, the sorted CD44^+^CD117^+^ cells were treated with metformin at 0.1 mM. The results showed that metformin treatment caused a 6.7-fold decrease in Survivin expression, and a 1.7-fold increase in Bax expression, without change in the Bcl-2 level (Fig. 4d). However, metformin did not affect the cell cycle activity of CD44^+^CD117^+^ cells (Fig. 4e).

### Metformin at low dose decreased CSCs and reduced EMT in SKOV3 xenograft mice

**Fig. 5:**
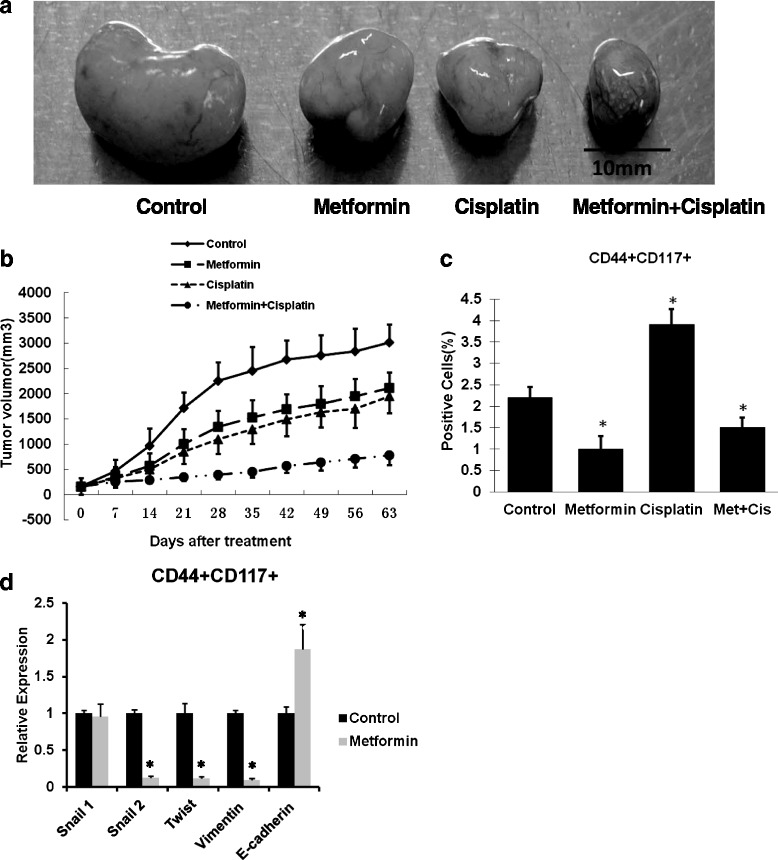
Metformin and cisplatin inhibit subcutaneous tumor growth in xenograft mice. Two to 4 weeks after subcutaneous injection of ovarian SKOV3 cancer cells, the treatments were started when the tumor size reached approximately 150 mm^3^. Four groups of mice (*n* = 6) were treated with saline, metformin, cisplatin, or the combination of metformin and cisplatin for 4 weeks. Tumor size and body weight were measured weekly throughout the experiment period. Three mice of each group were sacrificed after 4 weeks of treatment for analysis and the other three mice were kept to observe for an additional 5 weeks. Representative experiment showing the actual resected tumor of each treatment group (**a**). Summary results from three separate experiments are presented (**b**). After 4 weeks of treatment, tumor tissues from each group were digested with ultrapure collagenase IV to obtain single cell suspensions. Flow cytometry was performed to isolate CD44^+^CD117^+^ CSCs of each treatment group. Metformin treatment significantly reduced the CD44^+^CD117^+^ CSC population (**c**). Total RNA was isolated from CD44^+^CD117^+^cells and mRNA was quantitated by real-time RT-PCR. Metformin significantly reduced snail 2, twist, and vimentin, but not the snail1 expression level, and upregulated E-cadherin expression (**d**). * Significant difference compared with control (*P* < 0.05)

To further evaluate the therapeutic efficacy of metformin, human ovarian cisplatin-resistant SKOV3 tumor xenografts were used. Both metformin and cisplatin alone slightly inhibited tumor growth during 4 weeks of treatment (Fig. 5a, b). Following cessation of treatment, tumors in the metformin and cisplatin treatment groups grew less than the untreated tumors, demonstrating that treatment with metformin and cisplatin resulted in a delay of tumor growth. However, tumors grew much slower in mice treated with both metformin and cisplatin than after cessation of both treatments (Fig. 5b). FACS plots of sorted cells harvested from tumors in the different treatment groups demonstrate the effects on the CD44^+^CD117^+^ population (Fig. 5c). Cisplatin treatment resulted in an average increase of 77 % in the CD44^+^CD117^+^ population, likely because these cells are resistant to cell death from chemotherapy, similar to the enrichment observed in pancreatic cancer ALDH^+^-expressing cells following cisplatin treatment [6]. Treatment with metformin resulted in a 2.2-fold decrease in the CD44^+^CD117^+^ population, suggesting that metformin targeted the CSC population within the tumor. Combined treatment with metformin and cisplatin prevented the increase in the CSC population observed with cisplatin treatment alone, resulting in a 2.6-fold decrease in the CD44^+^CD117^+^ population compared with cisplatin treatment alone, which increased in the CSC population, further demonstrating that metformin is capable of targeting the CSC population (Fig. 5c). The effect of metformin on EMT markers’ expression of CD44^+^CD117^+^ cells was analyzed by real-time RT-PCR. Data demonstrated that 0.1 mM metformin had no effect on snail1 expression, but led to a 7.7-fold decrease for snail2, an 8.3-fold decrease for twist, a 10-fold decrease for vimentin, and a 1.9-fold increase for E-cadherin (Fig. 5d).

### Metformin at low dose decreased CSCs and reduced EMT in primary ovarian cancer xenograft mice

**Fig. 6:**
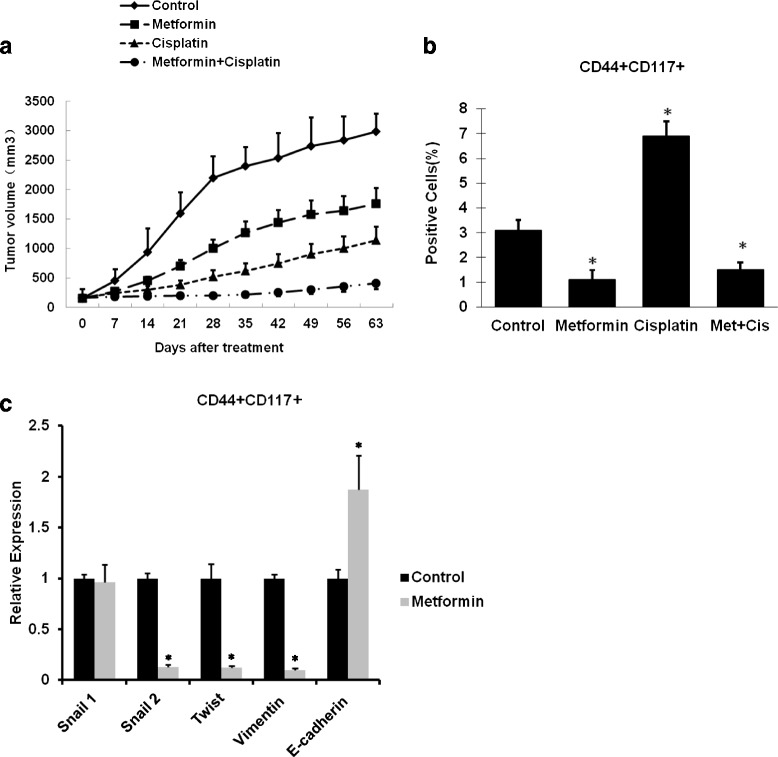
Metformin and cisplatin inhibit tumor growth in patient-derived xenograft mice. Six to 8 weeks after subcutaneous injection of ovarian patient ovarian cancer cells, the treatments were started when the tumor size reached approximately 150 mm^3^. Four groups of mice (*n* = 6) were treated with saline, metformin, cisplatin, and the combination of metformin and cisplatin for 4 weeks. Tumor size and body weight were measured weekly throughout the experiment period. Three mice from each group were sacrificed after 4 weeks of treatment for analysis and the other three mice were kept to observe for an additional 5 weeks. Summary results from three separate experiments are presented (**a**). Flow cytometry was performed to isolate CD44^+^CD117^+^ CSCs of each treatment group. Metformin treatment significantly reduced the CD44^+^CD117^+^ CSC population (**b**). Metformin significantly reduced snail2, twist, and vimentin, but not Snail1expression, and upregulated E-cadherin expression (**c**). * Significant difference compared with control (*P* < 0.05)

Data indicated that treatment with metformin or cisplatin alone resulted in a delay of tumor growth. However, in the group treated with metformin and cisplatin, tumor growth was extremely slow, even near the cessation of treatment, showing a significant enhanced anti-tumor effect (Fig. 6a). The effect of metformin on expression of EMT marker CD44^+^CD117^+^ was analyzed by real-time RT-PCR. We obtained similar results when measuring the effect of metformin, used alone or in combination with cisplatin, on the CD44^+^CD117^+^ CSC population (Fig. 6b). Treatment with metformin resulted in a 2.8-fold decrease in the CD44^+^CD117^+^ population, and an increase of 2.2-fold for cisplatin, and combined treatment with metformin and cisplatin resulted in a 4.6-fold decrease in the CD44^+^CD117^+^ population compared with cisplatin alone, and even a 2.1-fold decrease in the CD44^+^CD117^+^ population compared with control, further demonstrating that metformin is capable of targeting the CSC population (Fig. 6b). Real-time RT-PCR experiment demonstrated that metformin had no effect on Snail 1 expression, but led to a 11.1-fold decrease in Snail 2, a 10-fold decrease in Twist, a 11.1-fold decrease in Vimentin expression, and a 2.9-fold increase in E-cadherin expression (Fig. 6c).

## Discussion

In this study, we demonstrated that low concentrations of metformin selectively inhibited CD44^+^CD117^+^ ovarian CSCs through downregulation of EMT. Moreover, we showed that low concentrations of metformin downregulated snail2, twist, and vimentin, leading to inhibition of EMT, and downregulated Survivin expression to increase apoptosis in the CD44^+^CD117^+^ cell population. The efficacy study indicated that metformin also potentiated the effect of cisplatin. Therefore, low concentrations of metformin may be used as a therapeutic agent for killing ovarian CSCs.

Of clinical importance, CSCs have been hypothesized to be resistant to conventional chemotherapy and radiation therapy and to be responsible for cancer metastasis and recurrence after clinical remission. For ovarian cancer, CSCs are also believed to be resistant to chemotherapy and radiation therapy [23–25]. Metformin was active against ovarian cancer cells in vitro and in vivo. Also, metformin inhibited the growth of metastatic nodules in the lung and significantly potentiated cisplatin-induced cytotoxicity, resulting in approximately 90 % reduction in tumor growth in ovarian cancers [26]. Shank et al. [6] showed that metformin acted on ovarian CSCs, reducing the percentage of ALDH^+^ CSCs in vitro and in vivo, and inhibiting the growth of ovarian tumor spheres. In addition, metformin therapy alone slowed the growth of ovarian CSCs in vivo. Targeting Notch pathway of ovarian CSCs sensitized tumors to cisplatin therapy [27]. Low-dose metformin may reprogram ovarian cancer cells or CSCs into noncancerous cells [22]. These studies indicate that cisplatin treatment enriches the ovarian CSC population and that current treatments for ovarian cancer do not address the unique survival mechanisms of ovarian CSCs. It is therefore essential to better understand CSC so that specific therapies can be devised to target this population of cancer cells. Our in vivo data are consistent with these early findings, highlighting the significance of low-dose metformin in targeting CSCs.

Ovarian CSCs possess mesenchymal characteristics and EMT ability [28]. Ovarian CSCs could be a source of ovarian cancer metastasis through EMT and regulation of TWIST-1 expression, and function is a critical step in this process [29]. Ovarian CSCs that undergo the EMT have demonstrated that the tumor cells are in general less differentiable, more invasive, more chemoresistant, and result in poor clinical outcomes [29–31]. Chen et al. [9] demonstrated that the overexpression of miR-200c significantly reduced the CD117^+^CD44^+^ CSC xenograft growth and lung metastasis in vivo, partially through the reversal of the EMT phenotype. Snail1 and snail2 have also been associated with chemoresistance and activation of stemness-associated pathways [32, 33]. Craveiro et al. [34] showed that snail2 is one of the more significantly upregulated genes in paclitaxel-surviving CSCs and snail2 overexpression was sufficient to yield more aggressive and more chemoresistant tumors. Inhibition of the EMT process by snail1 silencing reduced the side population  cell frequency, and affected their invasive capacity and engraftment [35]. We therefore investigated the effect of metformin on EMT in CSCs, focusing on snail1, snail2, and twist, and established the connection between metformin and EMT of ovarian CSCs.

We firstly studied the effect of metformin at low concentration on the CSC population and found that low concentrations of metformin selectively inhibited CD44^+^CD117^+^ cells, but not CD133^+^ or ALDH^+^ cells, likely through downregulated EMT. We found that cisplatin could only kill the rapidly increased ovarian tumor cells, while it could not inhibit the population of CSCs. This is one of the reasons why many tumors are recurring and resistant to chemotherapy drugs, such as cisplatin. To study the effect of low concentrations of metformin on the function of CSCs, we utilized an in vitro sphere-forming assay. We found that using low concentrations of metformin decreased the CSC population and EMT. Our results are consistent with previous work indicating that low-dose metformin inhibits CSCs in other cancer types, such as pancreatic cancer and breast cancer [18, 21].

The underlying molecular mechanisms of metformin inhibiting EMT in CSCs are not well understood. Lee et al. [36] reported that induction of EMT in human retinal pigment epithelial cells upregulates Survivin, leading to Survivin-dependent inhibition of cell cycle arrest and apoptosis. It has been reported that drug resistance of ovarian CSCs were linked with Survivin [37]. Another study indicated that metformin induced apoptosis in ovarian cancer cells without significant cytotoxicity [38]. We found that metformin treatment resulted in apoptosis of ovarian CSCs by downregulating Survivin and upregulating Bax. These results suggest that low concentrations of metformin reduce survival of ovarian CSCs.

We analyzed the efficacy of targeting ovarian CSCs in xenografts of established ovarian cancers in nude mice and found that treatment with low-dose metformin in both SKOV3 and primary xenograft models resulted in a marked decrease in the ovarian CSC population. Both treatments with low-dose metformin alone or cisplatin alone did not considerably inhibit tumor growth, while treatment with both agents significantly blocked tumor growth, even up to 5 weeks after cessation of treatment. Low-dose metformin significantly inhibited CD44^+^CD177^+^ CSCs and EMT in vivo, which is consistent with our in vitro data. These data suggest that using a combined therapy that targets both the ovarian CSC population and the nontumorigenic bulk population of ovarian cancer cells may be most efficacious in treating patient symptoms associated with tumor mass and result in eradication of the CSC population.

As evidence mounts to support the paradigm that a small subset of cancer cells dictate the biologic behavior of malignant disease, approaches in designing more effective therapeutic strategies must consider a new target—the CSCs. Data have shown that ovarian cancers contain a small population of CSCs responsible for tumor initiation and propagation and resistance to conventional chemotherapy and radiation. Here we found that targeting low concentrations of metformin may be useful in eradicating CSCs and potentiate conventional chemotherapy. This study provides data to support further exploration of low concentrations of metformin, as a therapeutic target to eradicate CSCs from human ovarian tumors, which may prevent ovarian cancer recurrence and improve long-term survival.

## Conclusions

This research indicates that low concentrations of metformin selectively inhibit CD44^+^CD117^+^ ovarian CSCs through inhibitions of EMT and potentiate the effect of cisplatin. The present study may support the new clinical applications of metformin, which will prevent ovarian cancer recurrence and will improve long-term survival.

## Abbreviations

ALDH: Aldehyde dehydrogenase
APC: Allophycocyanin
CSC: Cancer stem cell
DAPI: 4′,6-Diamidino-2-phenylindole
DEAB: Diethylaminobenzaldehyde
EMT: Epithelial–mesenchymal transition
EOC: Epithelial ovarian cancer
FBS: Fetal bovine serum
HBSS: Hanks’ buffered salt solution
i.p.: Intraperitoneally
PBS: Phosphate-buffered saline
